# The neutrophil to lymphocyte ratio associates with markers of Alzheimer’s disease pathology in cognitively unimpaired elderly people

**DOI:** 10.21203/rs.3.rs-4076789/v1

**Published:** 2024-03-14

**Authors:** Tovia Jacobs, Sean R. Jacobson, Juan Fortea, Jeffrey S. Berger, Alok Vedvyas, Karyn Marsh, Tianshe He, Eugenio Gutierrez-Jimenez, Nathanael R. Fillmore, Omonigho M. Bubu, Moses Gonzalez, Luisa Figueredo, Naomi L. Gaggi, Chelsea Reichert Plaska, Nunzio Pomara, Esther Blessing, Rebecca Betensky, Henry Rusinek, Henrik Zetterberg, Kaj Blennow, Lidia Glodzik, Thomas M Wisniewski, Mony J. Leon, Ricardo S. Osorio, Jaime Ramos-Cejudo

**Affiliations:** New York University (NYU) Grossman School of Medicine; New York University (NYU) Grossman School of Medicine; Hospital de la Santa Creu y Sant Pau, Universitat Autònoma de Barcelona; NYU Grossman School of Medicine; New York University (NYU) Grossman School of Medicine; New York University (NYU) Grossman School of Medicine; New York University (NYU) Grossman School of Medicine; Aarhus University; MAVERIC, Boston Healthcare System; New York University (NYU) Grossman School of Medicine; New York University (NYU) Grossman School of Medicine; New York University (NYU) Grossman School of Medicine; New York University (NYU) Grossman School of Medicine; New York University (NYU) Grossman School of Medicine; New York University (NYU) Grossman School of Medicine; New York University (NYU) Grossman School of Medicine; New York University (NYU) Grossman School of Medicine; New York University (NYU) Grossman School of Medicine; University of Gothenburg; University of Gothenburg; Weill Cornell Medicine; New York University (NYU) Grossman School of Medicine; New York University (NYU) Grossman School of Medicine; New York University (NYU) Grossman School of Medicine; NYU Grossman School of Medicine

**Keywords:** NLR, Neutrophil to lymphocyte ratio, CSF, T-tau, P-tau, Amyloid-β, Alzheimer’s disease

## Abstract

**Background:**

An elevated neutrophil-lymphocyte ratio (NLR) in blood has been associated with Alzheimer’s disease (AD). However, an elevated NLR has also been implicated in many other conditions that are risk factors for AD, prompting investigation into whether the NLR is directly linked with AD pathology or a result of underlying comorbidities. Herein, we explored the relationship between the NLR and AD biomarkers in the cerebrospinal fluid (CSF) of cognitively unimpaired (CU) subjects. Adjusting for sociodemographics, APOE4, and common comorbidities, we investigated these associations in two cohorts: the Alzheimer’s Disease Neuroimaging Initiative (ADNI) and the M.J. de Leon CSF repository at NYU. Specifically, we examined associations between the NLR and cross-sectional measures of amyloid-β42 (Aβ42), total tau (t-tau), and phosphorylated tau_181_ (p-tau), as well as the trajectories of these CSF measures obtained longitudinally.

**Results:**

A total of 111 ADNI and 190 NYU participants classified as CU with available NLR, CSF, and covariate data were included. Compared to NYU, ADNI participants were older (73.79 vs. 61.53, p < 0.001), had a higher proportion of males (49.5% vs. 36.8%, p = 0.042), higher BMIs (27.94 vs. 25.79, p < 0.001), higher prevalence of hypertensive history (47.7% vs. 16.3%, p < 0.001), and a greater percentage of Aβ-positivity (34.2% vs. 20.0%, p = 0.009). In the ADNI cohort, we found cross-sectional associations between the NLR and CSF Aβ42 (β=−12.193, p = 0.021), but not t-tau or p-tau. In the NYU cohort, we found cross-sectional associations between the NLR and CSF t-tau (β = 26.812, p = 0.019) and p-tau (β = 3.441, p = 0.015), but not Aβ42. In the NYU cohort alone, subjects classified as Aβ+ (n = 38) displayed a stronger association between the NLR and t-tau (β = 100.476, p = 0.037) compared to Aβ− subjects or the non-stratified cohort. In both cohorts, the same associations observed in the cross-sectional analyses were observed after incorporating longitudinal CSF data.

**Conclusions:**

We report associations between the NLR and Aβ42 in the older ADNI cohort, and between the NLR and t-tau and p-tau_181_ in the younger NYU cohort. Associations persisted after adjusting for comorbidities, suggesting a direct link between the NLR and AD. However, changes in associations between the NLR and specific AD biomarkers may occur as part of immunosenescence.

## Background

Alzheimer’s disease (AD), the most common form of dementia, is characterized by amyloid-β (Aβ) plaques, neurofibrillary tangles (NFTs), and neuronal death (1). Biomarkers of these pathological features can be detected in the brain through neuroimaging such as magnetic resonance imaging (MRI) or positron emission tomography (PET), as well as in the cerebrospinal fluid (CSF) and blood. With these modalities, the National Institute on Aging and Alzheimer’s Association (NIA-AA) proposed an Aβ/tau/neurodegeneration (A/T/N) framework, and more recently inflammation/vascular/α-synuclein (I/V/S), to standardize the evaluation of biomarkers in AD research (2). However, variability in AD progression and cognitive outcomes of individuals classified using the A/T/N framework, coupled with substantial evidence from genome-wide association studies (GWAS) implicating immune system genes in AD development, suggests an important role of immune response mechanisms in the pathophysiology of AD (3–6).

Recent research has found neutrophils in the brains of AD patients, particularly near Aβ plaques, as well as increased numbers in the peripheral blood, suggesting a role of the innate immune response and systemic inflammation in AD progression (7–9). Key players in the adaptive immune response have also been suggested in AD, with elevated lymphocyte levels observed near Aβ plaques and tau aggregates in the brain, and variations in lymphocyte phenotypes reported in the CSF and peripheral blood (10–15). Alterations in the levels of peripheral cytokines involved in the innate and adaptive immune response have also been reported in AD patients, and some studies have even linked cytokine levels with the rate of cognitive decline in AD, including TNF-α, IFN-γ, and IL-10 (16–18). The neutrophil-lymphocyte ratio (NLR) in blood, often used as a general indicator of the balance between systemic inflammation and the adaptive immune response, has been associated with AD pathology, with elevated ratios seen in individuals with AD dementia and mild cognitive impairment (MCI) due to AD (19–22). Prior epidemiological research has also shown an association between the NLR and incident dementia risk in the elderly population (23). However, previous studies investigating the NLR in relation to AD/MCI have reported variable findings with some showing associations that failed to persist after adjusting for APOE4 and sociodemographic information (24, 25), while others showed associations that persisted after these adjustments (19, 20, 22, 26). Relatedly, previous studies were generally focused on end-stage disease, where it is challenging to disentangle the effects of age and comorbid chronic conditions known to increase the NLR in addition to being risk factors for AD (e.g., obesity, diabetes, hypertension) (27–40). Moreover, few studies have focused on analyzing the NLR in specific relation to amyloid and tau pathology, and none have had this focus within CU subjects while accounting for comorbidities.

The aim of our study was to investigate the associations between the NLR and AD biomarkers in a preclinical population, and to compare associations before and after comprehensive adjustment for comorbidities. In accordance with the A/T/N research framework, associations with Aβ were assessed via CSF Aβ42, with tau via CSF phosphorylated tau at threonine 181 (p-tau), and with neurodegeneration via CSF total tau (t-tau) (2). The study analyzed data from two distinct cohorts: the Alzheimer’s Disease Neuroimaging Initiative (ADNI), and the M.J. de Leon CSF repository at New York University (NYU), which is among the most extensive preclinical AD datasets with longitudinal complete blood count (CBC) and CSF data. We conducted a two-fold investigation examining: 1) cross-sectional associations between the baseline (first visit) NLR and baseline CSF biomarker measures, and 2) associations between the baseline NLR and longitudinal CSF biomarker measures obtained throughout follow-up visits. Further, since it has been reported that age-related increases in CSF p-tau and t-tau are dependent on Aβ burden in the preclinical setting (41–43), we subsequently examined these associations among participants categorized as Aβ− or Aβ+ (based on CSF values) to investigate potential interrelatedness between the NLR, amyloid burden, and p-tau and t-tau outcomes.

## Materials and Methods

### Study Population

#### ADNI

The ADNI cohort data included in this study were obtained from the study website (https://adni.loni.usc.edu/). The ADNI study was launched in 2003 as a public-private partnership, led by Principal Investigator Michael W. Weiner, MD. The primary focus of ADNI has been to track cognitive impairment progression and AD onset through a longitudinal study cohort while collecting clinical, biochemical, genetic, and imaging data. Recruited participants were between the ages of 55 and 90 years with cognitive statuses of cognitively unimpaired (CU), mild cognitive impairment (MCI), and AD. One CBC test was conducted for each subject as part of screening procedures, but subjects underwent additional biofluid testing, including CSF samples, during baseline and follow-up visits. Subjects included in this study were from the first three ADNI waves: ADNI1, ADNIGO, and ADNI2. The ADNI is a multisite program that abides by a standard protocol, and each site involved in data collection received local Institutional Review Board (IRB) approval. Written informed consent was obtained from enrolled subjects. Updated study information is available on the ADNI website.

#### NYU

The NYU cohort data included in this study were derived from multiple NIH-supported longitudinal studies spanning from 1996 to 2016, conducted under Principal Investigator Dr. Mony J de Leon. All studies shared the goal of identifying CSF biomarkers and MRI predictors of cognitive impairment in aging, and they included a standard protocol of medical, neurological, psychiatric, and neuropsychological testing in addition to clinical laboratory work, neuroimaging, and AD biomarker assessments. All studies were approved by the NYU Grossman School of Medicine Institutional Review Board (IRB), and written informed consent was obtained from each participant.

#### Study Criteria

Participants from the ADNI and NYU cohorts met study criteria if they were given a clinical diagnosis of CU at the time of CBC collection and had at least one accessible account of neutrophil and lymphocyte measures, demographic information, BMI, and APOE4 status, as well as medical histories indicating the presence/absence of Type 2 diabetes mellitus (T2DM) and hypertension. Subjects were also required to have undergone a CSF exam with accessible measures of Aβ42, t-tau, and p-tau181, considered only if CBC and CSF exams were collected concurrently. Outliers with CSF measures taken over 500 days from the baseline assessments were not considered in the analysis, as adjustments were based on baseline values.

#### CBC Clinical Lab Measurements

In the ADNI study, CBC exams were conducted during study screening visits for all participants, which took place a maximum of 28 days before baseline visits. Subjects were not required to fast before this blood draw. All blood collection vials were placed on dry ice and shipped for analysis the day of collection. Details on the ADNI methodology can be found on the ADNI website (http://www.adni-info.org/).

In the NYU cohort, CBC exams were conducted at baseline. Blood draws were collected in a fasting state. Samples were delivered for laboratory analysis immediately after collection.

NLR values for both cohorts were defined as the ratio of absolute neutrophils to absolute lymphocytes in the blood as determined by the CBC panel.

#### Vascular Risk Factors

In the ADNI study, history of hypertension was assessed during screening as part of the Modified Hachinski Ischemic Scale. Accordingly, the presence of hypertensive history was defined by a blood pressure >150/95 for at least 6 months prior to the screen. The presence of Type 2 diabetes mellitus (T2DM) was defined by self-reported diagnosis during medical history evaluations

In the NYU cohort, the presence of hypertensive history was defined by physical examination, a self-reported prior diagnosis, and documented use of hypertension medication. The presence of T2DM was defined by laboratory testing, a self-reported diagnosis, and medication history.

In both cohorts, body mass index (BMI) was computed as [weight (pounds) × 703] / height^2^ (inches).

#### CSF Measurements

In the ADNI study, CSF samples were collected for each participant at baseline as well as during select follow-ups. Lumbar punctures (LPs) were performed after a minimum 6 hours fast, and samples were immediately placed on dry ice and shipped overnight to the University of Pennsylvania Medical Center’s ADNI Biomarker Core Laboratory for analysis. The samples were run on the multianalyte Luminex xMAP platform using INNO-BIA AlzBio3 immunoassay reagents to detect Aβ42, t-tau and p-tau concentrations simultaneously (44). Longitudinal CSF collections were scaled to baseline. More information regarding CSF collection and analysis is discussed in the ADNI procedures manuals for each wave (http://www.adni-info.org/).

In the NYU cohort, CSF samples were collected at baseline and during select follow-ups. All LPs, CSF sample collections, and analyses were conducted in accordance with the recommendations of Vanderstichele et al. (2012) as they have been previously described (45, 46). In summary, LPs were performed between 10am-12pm after an overnight fast and a light breakfast. Concentrations of Aβ42, t-tau, and p-tau were obtained using INNOTEST sandwish enzyme-linked immunosorbent assays (ELISA). For Aβ42, batch-wise rescaling to a reference batch was conducted through linear regression, which reduced the coefficient of variation from 20% to 10%. No rescaling was conducted for t-tau or p-tau, which had coefficients of variation of 9% between batches.

### Statistical Analyses

R 4.2.2 was utilized for all statistical analyses and data visualization. Linear Mixed Models (LMM) were conducted using the *lmerTest* package (47). Comparative descriptive tables, utilizing Pearson’s chi-squared test for categorical variables and one-way ANOVA for continuous variables, were generated with the *tableone* package (48). Graphical figures, including linear trendlines and standard errors, were created using the *ggplot2* package (49).

The analyses involved the following variables: (a) NLR as an independent variable; (b) the three CSF biomarker measures as the outcome variables; and (c) demographic and clinical parameters, including age, sex, education, race, APOE4 status, time between CBC and CSF measurements, diabetes, BMI, and history of hypertension as covariates. A univariate model with the NLR as the only covariate was also constructed as part of each analysis in order to compare associations pre-and post-adjustment.

Generalized Linear Models (GLM) were used for the cross-sectional analyses, while LMM were utilized to explore baseline NLR associations with longitudinal CSF data. Accordingly, a time variable was implemented into the LMM independently and as an interaction variable with the NLR to determine variability over time. Random intercepts were also modeled for each subject in order to account for variation in baseline levels of the CSF outcomes and allow for individual-specific deviations from the overall model intercept. Lastly, mixed models were also adjusted to account for participants who were CU at CBC collection but experienced cognitive decline throughout the longitudinal collection of CSF data. This variable was included as a binary indicator in the models.

For the subset analyses based on amyloid status, the categorization of positivity was determined based on CSF values using a cutoff threshold of Aβ42 <192 pg/mL in the ADNI cohort, as recommended by Shaw et al. (2009) (50), and a cutoff threshold of Aβ42 <469.5 pg/mL in the NYU cohort, an internal value derived to distinguish between CU and AD diagnoses.

For sensitivity purposes and to integrate the data from both cohorts, a p-value meta-analysis using Stouffer’s method was conducted to obtain an overall assessment of statistical significance and generalizability. Stouffer’s method utilizes only p-values, sample sizes, and estimated directions to compute overall significance, so this method enabled an integrated assessment despite different CSF biomarker assays and detection methods between cohorts. For the purposes of this study, we defined statistical significance as p<0.050. All reported p-values are two-tailed.

## Results

### Population Characteristics

**Figure 1:**
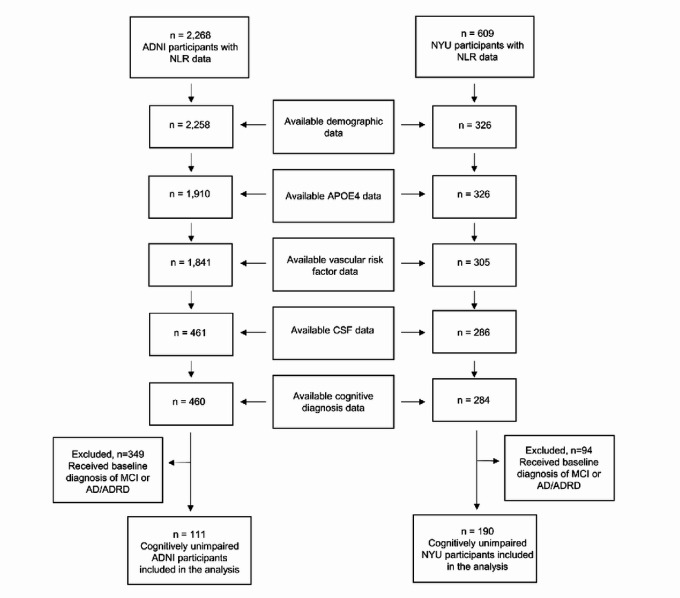
Study inclusion criteria. All subjects included in the analysis obtained a CBC exam at baseline, from which the NLR was calculated. Subjects were then filtered based on the availability of clinical, demographic, and CSF data collected during baseline visits in addition to receiving a formal baseline diagnosis of cognitively unimpaired (CU). Outliers with CSF measures taken over 500 days from the CBC exam were not considered.

A total of 111 ADNI participants and 190 NYU participants were included in the study, all of whom were defined as CU at baseline (Figure 1). Demographic and clinical characteristics at the baseline visit, stratified by cohort, are outlined in Table 1. Compared to the NYU cohort, the ADNI cohort was older (73.79 vs. 61.53, p<0.001), had a higher proportion of males (49.5% vs. 36.8%, p=0.042), higher BMIs (27.94 vs. 25.79, p<0.001), higher prevalence of hypertensive history (47.7% vs. 16.3%, p<0.001), and a greater percentage of Aβ-positivity (34.2% vs. 20.0%, p=0.009). The mean number of days between baseline CBC and CSF exams was 81.73 in the ADNI cohort and 28.33 in the NYU cohort (p<0.001).

The same characteristics, stratified by the median NLR at baseline for each cohort, can be found in **Supplementary Table 1** (ADNI) and **Supplementary Table 2** (NYU). In the ADNI cohort alone, subjects in the upper median NLR at baseline demonstrated a significantly greater prevalence of cognitive decline throughout longitudinal visits with CSF exams, compared to the lower median (1.8% vs. 18.2%, p=0.011).

After incorporating longitudinal CSF data (ADNI, n=274 data points; NYU, n=346), the mean number of follow-up visits was 2.47 in the ADNI cohort and 1.82 in the NYU cohort. The median time between CSF follow-up exams for a given subject, in years, was 1.03 [0.99, 1.95] within the ADNI cohort and 2.07 [1.75, 2.50] within the NYU cohort.

### Associations between the NLR and CSF Markers

#### ADNI Cohort

**Figure 2:**
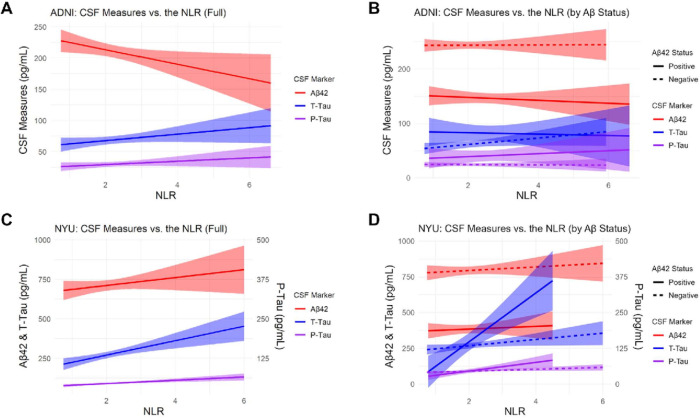
Baseline associations between the CSF markers and the NLR. CSF measures of Aβ42, P-Tau, and T-Tau were plotted vs the NLR in the: **A**) Full ADNI cohort. **B**) Aβ vs Aβ+ subjects in the ADNI cohort. C) Full NYU cohort. D) Aβ+ vs Aβ− subjects in the NYU cohort. Aβ positivity was defined by the predetermined CSF cut-off values for each cohort (NYU, <469.5 pg/mL; ADNI, <192 pg/mL).

In the cross-sectional analysis of the ADNI cohort (Table 2), baseline NLR inversely correlated with baseline Aβ42 levels in the univariate model (p=0.029) and after adjustment (p=0.021). No associations of the NLR with t-tau or p-tau were observed. Upon partitioning the cohort into Aβ− and Aβ+ subjects, no associations were observed between the NLR and any of the baseline CSF measures in either subset (Table 3). Figure 2 represents the baseline associations between the CSF markers and the NLR in the full cohorts and stratified by amyloid positivity status.

**Figure 3:**
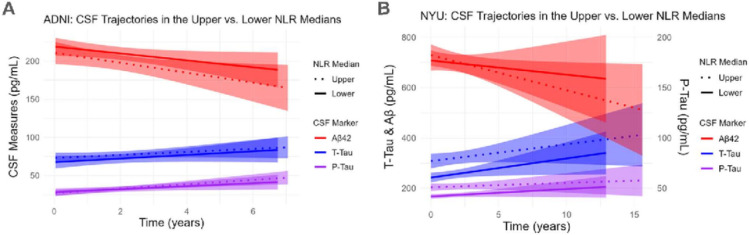
Longitudinal CSF trajectories of participants in the upper vs. lower median NLR at baseline. Linear trendlines were incorporated depicting the upper median (dotted line) and lower median (solid line) to highlight differences in trajectories of CSF measures of Aβ42, p-tau, and t-tau throughout follow-up visits. This was done for both cohorts: the ADNI (**A**) and NYU (**B**).

Similar results were obtained upon incorporating longitudinal CSF data (**Supplementary Table 3**). Baseline NLR inversely correlated with Aβ42 levels throughout follow-up exams in both the univariate model (p=0.020) and after adjustment (p=0.014). No associations with longitudinal measures of t-tau or p-tau were observed. Further, no associations were observed in the Aβ− nor Aβ+ subgroup in this analysis (**Supplementary Table 4**). The trajectories of CSF measures throughout follow-up visits in subjects of the lower vs. upper median NLR at baseline are depicted in Figure 3.

#### NYU Cohort

In the cross-sectional analysis of the NYU cohort, the baseline NLR positively associated with t-tau (p<0.001) and p-tau (p<0.001) levels in the univariate models as well as after adjustment (p=0.019, p=0.015, respectively) (Table 2). No associations of the NLR with Aβ42 were identified. After partitioning the cohort by amyloid status (Table 3), univariate associations with t-tau (p=0.040) and p-tau (p=0.017) were observed in the Aβ− subgroup, but these associations did not persist after adjustment. In the Aβ+ subgroup, univariate associations of the NLR with t-tau (p=0.001) and p-tau (p=0.002) were observed. In adjusted models, the association with t-tau was still significant (p=0.037), but this was not the case for p-tau. Compared to the results in the full cohort, the strength of the association of the NLR to t-tau and p-tau levels was greater in the Aβ+ subgroup (Table 3) (Figure 2).

When looking at longitudinal data, the baseline NLR positively correlated with longitudinal measures of t-tau and p-tau in the univariate models (p<0.001, p<0.001) as well as after adjustment (p=0.017, p=0.012, respectively) (**Supplementary Table 3**). No associations with Aβ42 were observed. In the Aβ− subgroup, univariate associations were observed between the NLR and both t-tau (p=0.045) and p-tau (p=0.014) measures throughout follow-up, but neither of these associations were observed post adjustment. In the Aβ+ subgroup, univariate associations were observed with t-tau (p<0.001) and p-tau (p=0.002), and the association with t-tau, but not p-tau, persisted post adjustment (p=0.020). Similar to the cross-sectional analysis, the strength of the association of NLR and t-tau and p-tau levels was greater in the Aβ+ subgroup compared to the full cohort (**Supplementary Table 4**). The trajectories of CSF measures throughout follow-up visits in subjects of the lower vs. upper median NLR at baseline are depicted in Figure 3.

#### Meta-Analysis

Results of the meta-analysis encompassing the full cross-sectional ADNI and NYU cohorts (**Supplementary Table 5**) indicated a positive association between the baseline NLR and t-tau and p-tau levels at baseline. This was observed in the univariate models (t-tau, p<0.001; p-tau, p<0.001) and after adjustment (t-tau, p=0.016; p-tau, p=0.012). A meta-analysis of Aβ− subjects from both cohorts showed a univariate association between the NLR and increased t-tau levels (p=0.014), but no additional associations with CSF outcomes were observed before or after adjustment. A meta-analysis of Aβ+ subjects from both cohorts, however, showed a univariate association between the NLR and increased p-tau levels (p=0.029), but no other associations with CSF outcomes were observed before or after adjustment.

The meta-analysis encompassing longitudinal CSF data from both cohorts (**Supplementary Table 5**) showed similar results, with the baseline NLR being associated with increased measures of t-tau and p-tau throughout follow-up in the univariate model (t-tau, p<0.001; p-tau, p=0.001) and after adjustment (t-tau, p=0.018; p-tau, p=0.011), while no association with Aβ42 was observed. A meta-analysis of Aβ− subjects from both cohorts showed a univariate association between the NLR and increased t-tau levels throughout follow-up (p=0.027), but no additional associations with CSF outcomes were observed before or after adjustment. Contrarily, a meta-analysis of Aβ+ subjects from both cohorts showed no associations between the NLR and any of the CSF measures throughout follow-up, although a univariate association was suggested for p-tau (p=0.051).

## Discussion

An elevated NLR, serving as an indicator of peripheral inflammation, has been implicated in AD pathology (19–22, 26). However, an elevated NLR has also been implicated in various other diseases and conditions, including those that are risk factors for AD, such as diabetes, hypertension, and obesity, raising questions as to whether the association with AD is simply a manifestation of underlying comorbidities (27–40). Further, the shortcoming of the NLR as a tool for the differential diagnosis with other dementias, due to its lack of specificity, has recently been highlighted (51). Therefore, the need for an investigation into the NLR in specific relation to Aβ and tau biomarkers, while accounting for comorbidities, has become apparent, particularly within the A/T/N framework. In this study, we report significant associations between the NLR and CSF Aβ42 in the CU ADNI cohort, as well as between the NLR and CSF p-tau_181_ and t-tau in the CU NYU cohort. These associations were observed before and after adjusting for sociodemographic information, APOE4, and common comorbidities (diabetes, hypertension, BMI).

The associations we report between the NLR and A/T/N characteristics in each cohort could be partially explained by recent research focused on neutrophil involvement in AD pathology. Neutrophils, the predominant form of leukocytes, play an important role in innate immunity by defending against pathogens and clearing cellular debris. However, their pro-inflammatory nature, including the release of reactive oxygen species (ROS), lytic enzymes, and neutrophil extracellular traps (NETs), can cause tissue damage through sustained exposure, largely explaining their implication in many inflammatory diseases (52–55). Although the brain was once considered an immune-privileged site, neutrophils and NETs have been shown to aggregate in the cerebral small vessels of AD patients, particularly near Aβ plaques and tau tangles (7, 8, 56). This phenomenon has been attributed to disruption of the blood-brain barrier (BBB) and the release of cytokines and chemokines by microglia in response to local amyloid plaques, leading to the recruitment of neutrophils into the brain parenchyma to help clear the misfolded protein aggregates (8, 56, 57). Indeed, the chronic presence of these neutrophils in the brain can lead to sustained neuroinflammation, which is pertinent considering hyperactive and advanced adhesive phenotypes such as elevated expression of the CD11b adhesion molecule have been observed in the neutrophils of AD patients (7, 8, 58, 59). Moreover, aging, which remains the greatest risk factor for AD, has been associated with neutrophil abnormalities including impaired chemotaxis and increased ROS release (60–62). Zanero et al. even reported an Aβ-induced transition of the neutrophil-arresting LFA-1 integrin to a higher affinity state, thereby perpetuating neutrophil adhesion in amyloid-rich regions (8). In the same studies, a reduction in cognitive impairment was reported after neutrophils were depleted or neutrophil trafficking was inhibited via LFA-1 integrin blockade. Taken together, these reports may support our findings, particularly in the ADNI cohort where an elevated NLR associated with Aβ brain deposition, and subjects in the upper median NLR at baseline demonstrated greater prevalence of subsequent cognitive decline. Previous research has also shown that pro-inflammatory cytokines released by activated microglia near amyloid plaques, including TNFα, IFNγ, and IL-1β, can increase Aβ peptide levels and decrease amyloid plaque clearance (63–66), promoting a positive feedback cycle between amyloid plaque deposition, microglial activation, neutrophil/immune cell recruitment, and neuroinflammation, which may further support our findings. The Aβ-induced microglial release of IL-1β, which is involved in neutrophil recruitment, has also been linked with increased BBB disruption and tau pathology in murine models (66–69), which may partially support our findings in the NYU cohort, where the NLR associated with both neurodegeneration and tau pathology. In addition to their recruitment into the brain, elevated levels and phenotypic changes of neutrophils in the peripheral blood of AD patients have been reported (9, 59), which may also support our findings in both cohorts. However, the discrepancies we report in specific associations between cohorts suggest the involvement of additional factors, and the role of lymphocytes is also relevant when considering the NLR in relation to AD and A/T/N.

Previous literature surrounding lymphocytes, leukocytes involved in the adaptive immune response, has highlighted their contribution to the pro-inflammatory milieu outlined above. Similar to neutrophils, lymphocytes, particularly CD4+ and CD8+ T-cells, have been observed in the brains of AD patients near microglia, suggesting dynamic cross-talk and interdependence on microglial phenotype, T-cell differentiation, and pro-inflammatory outcomes (10, 12, 13, 15). In general, CD4+ T-cells exhibit diverse roles in modulating microglia, with regulatory T-cells (Tregs) secreting anti-inflammatory cytokines and effector T-cells (Teffs) secreting pro-inflammatory cytokines. Although the anti-inflammatory benefits of Tregs in AD and their role in Aβ clearance has been highlighted (70), Teffs have been shown to progress AD pathology and even downregulate Tregs in later disease stages in murine models (71). Pro-inflammatory CD8+ T-cells have generally been observed in close proximity to tau aggregates in the AD brain, and their involvement in tauopathy has been suggested (10, 72). Although our study did not differentiate lymphocyte subsets, this may support our findings in the NYU cohort linking the NLR with tau pathology. Additionally, the pro-inflammatory response from T-cell activation in the brain has been linked with compromised BBB integrity and peripheral immune cell influx (73), with a study by Yang et al. suggesting an Aβ-induced release of TNFα by microglia to promote T-cell recruitment to the brain in AD (74), further feeding in to the aforementioned positive feedback loop. Contrarily, a study by Chen et al. demonstrated that microglial recruitment of T-cells in response to tauopathy, but not amyloid deposition, leads to neurodegeneration in AD (72), and a study by Merlini et al. reported CD3+ T-cells, most of which were CD8+, to correlate with tau, but not amyloid, pathology in the AD brain (10). Taken together, these studies may further support our findings in the NYU cohort which demonstrated associations between the NLR and both tau pathology and neurodegeneration, but not Aβ. Relevant to our findings in both cohorts, many studies have reported a decrease in the total count of lymphocytes in the peripheral blood in AD (although conflicting findings have also been reported), with mechanisms being attributed to influx in the CNS as well as susceptibility to ROS, which is pertinent given the observed increase in ROS released by neutrophils in AD as mentioned above. Regardless, an increase in the proportion of activated HLA-DR+ CD4+ and CD8+ T-cells has been observed in the peripheral blood of AD patients, even in studies which observed no difference in the total count (14, 75, 76).

These pathophysiological changes in AD in the context of both neutrophils and lymphocytes may partially explain why an elevated NLR has been associated with cortical Aβ deposition, as determined by PET and CSF Aβ measures, and a greater risk of cognitive decline in AD cohorts (19–22, 26). The associations we report between the NLR and decreased CSF Aβ42 in the ADNI cohort support these previous studies and suggests the associations are not simply an artifact of comorbidities that raise the NLR and pose as risk factors for AD. In particular, our results in the ADNI cohort support the study by Hou et al. which reported an association between the NLR and lower CSF Aβ42 in CU participants within the ADNI after adjusting for sociodemographics and APOE4 alone (26). Another study conducted in the ADNI cohort, by Mehta et al., that included MCI and AD participants in the analysis, reported an elevated NLR to associate with greater PET measures of Aβ, but not tau, as well as with longitudinal cognitive decline determine by the Alzheimer’s Disease Assessment Scale Cognitive Subscale (ADAS-Cog) (22). Our study supports these findings, demonstrating that the association between the NLR and Aβ deposition are present even within the strictly CU population in the ADNI cohort. Likewise, although we defined cognitive decline as the transition from CU to a clinical diagnosis of MCI or AD throughout follow-up visits, rather than by changes in the ADAS-Cog score alone, the increased incidence of cognitive decline that we report in the ADNI subjects who were in the upper median NLR at baseline supports the reported study. This association between the NLR and longitudinal cognitive decline also supports the study of Ramos-Cejudo et al. who found an increased NLR to associate with a greater risk of incident dementia in the elderly population after comprehensive adjustment (23). However, the associations between the NLR and p-tau and t-tau, but not Aβ42, that we report in the NYU cohort, which displayed a younger aging profile, may suggest the NLR associates with tau pathology and neurodegeneration, but not amyloid deposition, in earlier stages of the disease process.

Further, our results in the NYU cohort suggest the association between the NLR, tau pathology, and neurodegeneration may be greater in participants who are amyloid-positive. This may be due to greater BBB disruption resulting in increased immune cell recruitment into the CNS by microglia-released pro-inflammatory cytokines, whether neutrophil recruitment via IL-1β or T-cell recruitment via TNFα, as outlined in the studies mentioned above. This may also be supported by the findings of Zhang et al. who demonstrated increased T-cell recruitment into the brain parenchyma as a result of BBB disruption in Aβ-induced AD rats (73). Another possible mechanism may be explained by Man et al. who suggested amyloid-induced overexpression of macrophage inflammatory protein-1 alpha (MIP-1α) in peripheral T-cells of AD patients to promote their migration across the BBB (77). Regardless of mechanism, our findings of potential amyloid-mediated associations between the NLR, tau pathology, and neurodegeneration may be supported by Ising et al. who demonstrated that activation of the NLRP3 inflammasome mediates Aβ-induced tau pathology in the AD mouse model (69). Additionally, Bellaver et al. reported Aβ-positivity to associate with tau tangle accumulation in CU individuals only if they were positive for astrocyte reactivity based on their plasma glial fibrillary acidic protein (GFAP) levels (78). Future studies could therefore benefit from incorporating GFAP data in addition to the NLR to explore potential interplay in amyloid and tau outcomes. Another study by Rabin et al. reported that cerebral amyloid angiopathy interacted with neuritic Aβ plaques to promote cognitive decline, and this interaction was mediated by tau, which may support our findings of a dynamic interplay between peripheral inflammation, amyloid burden, and tau pathology (79). Lastly, Milà-Alomà et al. reported that changes in CSF p-tau and t-tau were associated with age in Aβ+ subjects only (80). Although our study suggests changes in CSF t-tau are associated with the NLR, not age, in these subjects, it supports the notion that Aβ-induced changes in tau pathology (41–43, 78, 80, 81) are mediated by additional factors. To our knowledge, we are the first to report an association between the NLR and CSF measures of t-tau and p-tau in the preclinical setting, a dynamic relationship between peripheral immunity, tau pathology, and neurodegeneration that may be more pronounced in the presence of amyloid deposition.

In understanding the disparities in specific biomarker associations between cohorts, the statistically significant variations in cohort characteristics, including age, amyloid burden, hypertensive histories, and BMIs are likely contributors. With the older ADNI cohort demonstrating a greater presence of age-related conditions, our results suggest that the general influence of aging on the immune system, or immunosenescence, may contribute to the varied predictive capabilities of the NLR across cohorts of distinct immune profiles. Propensity score matching between the two groups could not be conducted to address these considerations due to the relatively small sample sizes and drastic cohort differences leading to inadequate match results. Similarly, although the meta analysis encompassing both cohorts showed associations between an elevated NLR and increases in both t-tau and p-tau after adjustment, further suggesting an association between the NLR and both tau pathology and neurodegeneration, associations between the NLR and Aβ42 could not be adequately assessed due to directional differences in the β coefficients between cohorts, once again highlighting the variability of specific associations between the NLR and A/T/N markers dependent on immunity and aging profiles. Therefore, future studies could benefit from conducting an extensive longitudinal analysis encompassing the age ranges of both cohorts in our study to investigate how age plays a factor in the interplay between the NLR and these CSF markers, and how associations with specific markers in the A/T/N framework may change as a result of immunosenescence.

In the same light, additional comorbidities which have been shown to increase the NLR, such as depression, cancer, and cardiovascular diseases, may be confounders (82–86), and a limitation of our study was the inability to account for these variables due to the absence of data and sample size limitations. Future studies conducting similar investigations should consider adjusting for these additional comorbidities as well. Future studies should also consider assessing the NLR as a risk factor for cognitive decline to AD or MCI due to AD by means of survival analysis, an analysis we could not explore due to sample size limitations and the absence of longitudinal CBC data in the ADNI. Although we report greater prevalence of cognitive decline in the ADNI subjects within the upper median NLR at baseline, a survival analysis leveraging longitudinal NLR, comorbidity, and A/T/N framework data could be more beneficial in highlighting specific pathways in disease progression. Another limitation of our study was that we investigated associations with AD biomarkers in the CSF in order to maximize sample size, but future studies could benefit from incorporating PET and MRI data, which may be more accurate at assessing structural and functional changes within the A/T/N and I/V/S frameworks. In fact, the absence of associations between the NLR and t-tau or p-tau among Aβ+ subjects in the ADNI cohort may be partially explained by the findings of Reimand et al. who reported that participants in the ADNI who met the Aβ-positivity threshold in both the CSF and PET had substantially greater levels of tau after 5 years when compared to those who met the Aβ-positivity threshold in the CSF but not PET (87). Therefore, a limitation of our study was defining Aβ-positivity by CSF levels, which may define an earlier and less understood level of amyloid deposition, and future studies could benefit from conducting the same analysis using the amyloid PET cutoff threshold instead. Future studies could also benefit from incorporating p-tau231 and p-tau217 measures, as they have been more closely associated with Aβ deposition in previous studies, compared to p-tau_181_, even in early stages prior to significant Aβ plaque buildup (80, 81). Lastly, a limitation of our study included differences in variable determination and collection procedures between cohorts. For example, only the NYU cohort included the documented use of hypertensive medications as a requirement in defining history of hypertension, so future studies could benefit from incorporating a standardized definition or raw blood pressure data.

## Conclusion

Our study found the NLR to independently associate with AD biomarkers in CU subjects even after comprehensive adjustment for sociodemographics, APOE4, and common comorbidities. The NLR was associated with lower CSF Aβ42 values in the ADNI cohort, which was significantly older and characterized by greater vascular risk compared to the NYU cohort. The NLR was associated with CSF p-tau_181_ and t-tau in the younger NYU cohort. In the NYU cohort alone, the association between the NLR and t-tau was much stronger among Aβ+ subjects. The associations in both cohorts were consistent after incorporating longitudinal trajectories of CSF values. Our results suggest that changes in the associations between the NLR and specific AD-biomarkers may occur as part of immunosenescence, which should be further examined by including more age-related and comorbidity-influenced measures.

## Figures and Tables

**Table 1:** Participant Characteristics at Baseline

	ADNI	NYU	p	
n	111	190		
Age (Mean, SD)	73.79 (6.43)	61.53 (10.94)	<0.001	***
Sex (m) (n, %)	55 (49.5)	70 (36.8)	0.042	*
Education (Mean, SD)	16.25 (2.74)	16.75 (2.14)	0.082	
Race (white) (n, %)	95 (85.6)	174 (91.6)	0.152	
APOE4 (n, %)	32 (28.8)	58 (30.5)	0.857	
Days Between Exams (Mean, SD)	81.73 (99.10)	28.33 (71.93)	<0.001	***
BMI (Mean, SD)	27.94 (4.65)	25.79 (4.39)	<0.001	***
History of Hypertension (n, %)	53 (47.7)	31 (16.3)	<0.001	***
Diabetic (n, %)	2 (1.8)	5 (2.6)	0.949	
Cognitive Decline (n, %)	11 (9.9)	8 (4.2)	0.086	
CSF Aβ Positive (n, %)	38 (34.2)	38 (20.0)	0.009	**
CSF Aβ (Mean, SD)*	211 (55)	714 (232)	NA	
CSF T-Tau (Mean, SD)*	69 (33)	275 (146)	NA	
CSF P-Tau (Mean, SD)*	30 (21)	46 (18)	NA	
NLR (Mean, SD)	2.23 (0.98)	2.09 (0.87)	0.209	

All included subjects had complete accounts of the above data. Categorical variable differences were calculated using chi-squared tests, and continuous variable differences were calculated using ANOVA tests. APOE4 positivity was defined by the presence of at least one APOE4 allele. CSF Aβ positivity was defined by the respective cut-off threshold for each cohort (NYU, <469.5 pg/mL; ADNI, <192 pg/mL). Days Between Exams was calculated as the number of days between the cross-sectional CSF exam and CBC exam from which the NLR was calculated. Cognitive decline refers to subjects who were given a diagnosis of MCI or AD during follow-up CSF collection, and this binary variable was included as an additional adjustment in the longitudinal analysis.

* CSF measures were obtained and scaled using separate procedures for each cohort, including the use of different antibody assays, so cohort differences could not be calculated (see Methods).

**Table 2:** Cross-Sectional Associations between the NLR and CSF Outcomes in the ADNI and NYU Cohorts

		Univariate Model		Adjusted Model	
Cohort	CSF Outcome	NLR β	NLR p-value	NLR β	NLR p-value
**ADNI**	**Aβ42**	−11.623	**0.029**	−12.193	**0.021**
	**t-tau**	5.126	0.110	3.924	0.230
	**p-tau**	2.602	0.202	2.464	0.227
**NYU**	**Aβ42**	24.979	0.199	19.109	0.330
	**t-tau**	45.698	**<0.001**	26.812	**0.019**
	**p-tau**	5.211	**<0.001**	3.441	**0.015**

Unadjusted and adjusted linear regression models for the association of the NLR and the three outcome variables are shown (Aβ42, t-tau, p-tau). Adjusted models included age, sex, education, race, APOE4, time between CBC and CSF exams, BMI, history of hypertension, and diabetes. β and p-values are shown.

**Table 3:** Cross-Sectional Associations between the NLR and CSF Outcomes in the Amyloid Positive and Amyloid Negative Subsets of the ADNI and NYU Cohorts

ADNI Cohort	Aβ Negative (n=73)				Aβ Positive (n=38)			
	Univariate Model		Adjusted Model		Univariate Model		Adjusted Model	
CSF Outcome	β	p-value	β	p-value	β	p-value	β	p-value
Aβ42	0.312	0.934	−3.057	0.440	−2.640	0.558	2.145	0.682
t-tau	5.848	0.074	5.620	0.118	−1.473	0.825	−7.596	0.371
p-tau	−0.237	0.872	0.197	0.903	2.483	0.603	−0.204	0.974
NYU Cohort	Aβ Negative (n=152)				Aβ Positive (n=38)			
	Univariate Model		Adjusted Model		Univariate Model		Adjusted Model	
CSF Outcome	β	p-value	β	p-value	β	p-value	β	p-value
Aβ42	12.538	0.440	3.946	0.810	9.641	0.610	17.645	0.486
t-tau	21.980	**0.040**	12.015	0.249	171.399	**<0.001**	100.476	**0.037**
p-tau	3.279	**0.017**	2.453	0.083	15.105	**0.002**	8.037	0.138

Unadjusted and adjusted linear regression models for the association of the NLR and the three outcome variables are shown (Aβ42, t-tau, p-tau). Adjusted models included age, sex, education, race, APOE4, time between CBC and CSF exams, BMI, history of hypertension, and diabetes. Aβ positivity was defined by the CSF cut-off threshold for each cohort. β and p-values are shown.

## References

[1] As Association. 2023 Alzheimer’s disease facts and figures. Alzheimers Dement. 2023;19(4):1598–695. 36918389 10.1002/alz.13016

[2] Jack CR Jr., Bennett DA, Blennow K, Carrillo MC, Feldman HH, Frisoni GB, A/T/N: An unbiased descriptive classification scheme for Alzheimer disease biomarkers. Neurology. 2016;87(5):539–47. 27371494 10.1212/WNL.0000000000002923 PMC4970664

[3] Lorius N, Locascio JJ, Rentz DM, Johnson KA, Sperling RA, Viswanathan A, Marshall GA. Vascular disease and risk factors are associated with cognitive decline in the alzheimer disease spectrum. Alzheimer Dis Assoc Disord. 2015;29(1):18–25. 24787033 10.1097/WAD.0000000000000043 PMC4366000

[4] Jansen IE, Savage JE, Watanabe K, Bryois J, Williams DM, Steinberg S, Genome-wide meta-analysis identifies new loci and functional pathways influencing Alzheimer’s disease risk. Nat Genet. 2019;51(3):404–13. 30617256 10.1038/s41588-018-0311-9 PMC6836675

[5] Marioni RE, Harris SE, Zhang Q, McRae AF, Hagenaars SP, Hill WD, GWAS on family history of Alzheimer’s disease. Transl Psychiatry. 2018;8(1):99. 29777097 10.1038/s41398-018-0150-6 PMC5959890

[6] Kunkle BW, Grenier-Boley B, Sims R, Bis JC, Damotte V, Naj AC, Genetic meta-analysis of diagnosed Alzheimer’s disease identifies new risk loci and implicates Aβ, tau, immunity and lipid processing. Nat Genet. 2019;51(3):414–30. 30820047 10.1038/s41588-019-0358-2 PMC6463297

[7] Smyth LCD, Murray HC, Hill M, van Leeuwen E, Highet B, Magon NJ, Neutrophil-vascular interactions drive myeloperoxidase accumulation in the brain in Alzheimer’s disease. Acta Neuropathol Commun. 2022;10(1):38. 35331340 10.1186/s40478-022-01347-2 PMC8944147

[8] Zenaro E, Pietronigro E, Della Bianca V, Piacentino G, Marongiu L, Budui S, Neutrophils promote Alzheimer’s disease-like pathology and cognitive decline via LFA-1 integrin. Nat Med. 2015;21(8):880–6. 26214837 10.1038/nm.3913

[9] Luo J, Thomassen JQ, Nordestgaard BG, Tybjærg-Hansen A, Frikke-Schmidt R. Blood Leukocyte Counts in Alzheimer Disease. JAMA Netw Open. 2022;5(10):e2235648. 36215071 10.1001/jamanetworkopen.2022.35648 PMC9552891

[10] Merlini M, Kirabali T, Kulic L, Nitsch RM, Ferretti MT. Extravascular CD3+ T Cells in Brains of Alzheimer Disease Patients Correlate with Tau but Not with Amyloid Pathology: An Immunohistochemical Study. Neurodegener Dis. 2018;18(1):49–56. 29402847 10.1159/000486200

[11] Gate D, Saligrama N, Leventhal O, Yang AC, Unger MS, Middeldorp J, Clonally expanded CD8 T cells patrol the cerebrospinal fluid in Alzheimer’s disease. Nature. 2020;577(7790):399–404. 31915375 10.1038/s41586-019-1895-7 PMC7445078

[12] Unger MS, Li E, Scharnagl L, Poupardin R, Altendorfer B, Mrowetz H, CD8(+) T-cells infiltrate Alzheimer’s disease brains and regulate neuronal-and synapse-related gene expression in APP-PS1 transgenic mice. Brain Behav Immun. 2020;89:67–86. 32479993 10.1016/j.bbi.2020.05.070

[13] Jorfi M, Park J, Hall CK, Lin CJ, Chen M, von Maydell D, Infiltrating CD8(+) T cells exacerbate Alzheimer’s disease pathology in a 3D human neuroimmune axis model. Nat Neurosci. 2023;26(9):1489–504. 37620442 10.1038/s41593-023-01415-3 PMC11184920

[14] Lueg G, Gross CC, Lohmann H, Johnen A, Kemmling A, Deppe M, Clinical relevance of specific T-cell activation in the blood and cerebrospinal fluid of patients with mild Alzheimer’s disease. Neurobiol Aging. 2015;36(1):81–9. 25277040 10.1016/j.neurobiolaging.2014.08.008

[15] Togo T, Akiyama H, Iseki E, Kondo H, Ikeda K, Kato M, Occurrence of T cells in the brain of Alzheimer’s disease and other neurological diseases. J Neuroimmunol. 2002;124(1–2):83–92. 11958825 10.1016/s0165-5728(01)00496-9

[16] Holmes C, Cunningham C, Zotova E, Woolford J, Dean C, Kerr S, Systemic inflammation and disease progression in Alzheimer disease. Neurology. 2009;73(10):768–74. 19738171 10.1212/WNL.0b013e3181b6bb95 PMC2848584

[17] Leung R, Proitsi P, Simmons A, Lunnon K, Güntert A, Kronenberg D, Inflammatory proteins in plasma are associated with severity of Alzheimer’s disease. PLoS One. 2013;8(6):e64971. 23762274 10.1371/journal.pone.0064971 PMC3677891

[18] Motta M, Imbesi R, Di Rosa M, Stivala F, Malaguarnera L. Altered plasma cytokine levels in Alzheimer’s disease: correlation with the disease progression. Immunol Lett. 2007;114(1):46–51. 17949824 10.1016/j.imlet.2007.09.002

[19] Kuyumcu ME, Yesil Y, Oztürk ZA, Kizilarslanoğlu C, Etgül S, Halil M, The evaluation of neutrophil-lymphocyte ratio in Alzheimer’s disease. Dement Geriatr Cogn Disord. 2012;34(2):69–74. 22922667 10.1159/000341583

[20] Chou OHI, Zhou J, Li L, Chan JSK, Satti DI, Chou VHC, The Association Between Neutrophil-Lymphocyte Ratio and Variability with New-Onset Dementia: A Population-Based Cohort Study. J Alzheimers Dis. 2023;94(2):547–57. 37302029 10.3233/JAD-220111

[21] Kalelioglu T, Yuruyen M, Gultekin G, Yavuzer H, Özturk Y, Kurt M, Neutrophil and platelet to lymphocyte ratios in people with subjective, mild cognitive impairment and early Alzheimer’s disease. Psychogeriatrics. 2017;17(6):506–8. 28386987 10.1111/psyg.12260

[22] Mehta NH, Zhou L, Li Y, McIntire LB, Nordvig A, Butler T, Peripheral immune cell imbalance is associated with cortical beta-amyloid deposition and longitudinal cognitive decline. Sci Rep. 2023;13(1):8847. 37258519 10.1038/s41598-023-34012-2 PMC10232445

[23] Ramos-Cejudo J, Johnson AD, Beiser A, Seshadri S, Salinas J, Berger JS, The Neutrophil to Lymphocyte Ratio Is Associated With the Risk of Subsequent Dementia in the Framingham Heart Study. Front Aging Neurosci. 2021;13:773984. 34916927 10.3389/fnagi.2021.773984 PMC8670436

[24] Rembach A, Watt AD, Wilson WJ, Rainey-Smith S, Ellis KA, Rowe CC, An increased neutrophil-lymphocyte ratio in Alzheimer’s disease is a function of age and is weakly correlated with neocortical amyloid accumulation. J Neuroimmunol. 2014;273(1–2):65–71. 24907904 10.1016/j.jneuroim.2014.05.005

[25] Kara SP, Altunan B, Unal A. Investigation of the peripheral inflammation (neutrophil-lymphocyte ratio) in two neurodegenerative diseases of the central nervous system. Neurol Sci. 2022;43(3):1799–807. 34331157 10.1007/s10072-021-05507-5 PMC8324446

[26] Hou JH, Ou YN, Xu W, Zhang PF, Tan L, Yu JT. Association of peripheral immunity with cognition, neuroimaging, and Alzheimer’s pathology. Alzheimers Res Ther. 2022;14(1):29. 35139899 10.1186/s13195-022-00968-y PMC8830026

[27] Rodríguez-Rodríguez E, López-Sobaler AM, Ortega RM, Delgado-Losada ML, López-Parra AM, Aparicio A. Association between Neutrophil-to-Lymphocyte Ratio with Abdominal Obesity and Healthy Eating Index in a Representative Older Spanish Population. Nutrients. 2020;12(3). 10.3390/nu12030855 PMC7146141 32210070

[28] Zhao T, Zhong T, Zhang M, Xu Y, Zhang M, Chen L. Alzheimer’s Disease: Causal Effect between Obesity and APOE Gene Polymorphisms. Int J Mol Sci. 2023;24(17). 10.3390/ijms241713531 PMC10487910 37686334

[29] Lee S, Byun MS, Yi D, Kim MJ, Jung JH, Kong N, Body mass index and two-year change of in vivo Alzheimer’s disease pathologies in cognitively normal older adults. Alzheimers Res Ther. 2023;15(1):108. 37312229 10.1186/s13195-023-01259-w PMC10262499

[30] Mertoglu C, Gunay M. Neutrophil-Lymphocyte ratio and Platelet-Lymphocyte ratio as useful predictive markers of prediabetes and diabetes mellitus. Diabetes Metab Syndr. 2017;11 Suppl 1:S127–s31. 28017281 10.1016/j.dsx.2016.12.021

[31] Demirtas L, Degirmenci H, Akbas EM, Ozcicek A, Timuroglu A, Gurel A, Ozcicek F. Association of hematological indicies with diabetes, impaired glucose regulation and microvascular complications of diabetes. Int J Clin Exp Med. 2015;8(7):11420–7. 26379958 PMC4565341

[32] Lou M, Luo P, Tang R, Peng Y, Yu S, Huang W, He L. Relationship between neutrophil-lymphocyte ratio and insulin resistance in newly diagnosed type 2 diabetes mellitus patients. BMC Endocr Disord. 2015;15:9. 25887236 10.1186/s12902-015-0002-9 PMC4357061

[33] Luchsinger JA, Reitz C, Patel B, Tang MX, Manly JJ, Mayeux R. Relation of diabetes to mild cognitive impairment. Arch Neurol. 2007;64(4):570–5. 17420320 10.1001/archneur.64.4.570

[34] Roberts RO, Knopman DS, Geda YE, Cha RH, Pankratz VS, Baertlein L, Association of diabetes with amnestic and nonamnestic mild cognitive impairment. Alzheimers Dement. 2014;10(1):18–26. 23562428 10.1016/j.jalz.2013.01.001 PMC3830601

[35] Liu X, Zhang Q, Wu H, Du H, Liu L, Shi H, Blood Neutrophil to Lymphocyte Ratio as a Predictor of Hypertension. Am J Hypertens. 2015;28(11):1339–46. 25824450 10.1093/ajh/hpv034

[36] Jhuang YH, Kao TW, Peng TC, Chen WL, Li YW, Chang PK, Wu LW. Neutrophil to lymphocyte ratio as predictor for incident hypertension: a 9-year cohort study in Taiwan. Hypertens Res. 2019;42(8):1209–14. 30850753 10.1038/s41440-019-0245-3 PMC8075864

[37] Glodzik L, Mosconi L, Tsui W, de Santi S, Zinkowski R, Pirraglia E, Alzheimer’s disease markers, hypertension, and gray matter damage in normal elderly. Neurobiol Aging. 2012;33(7):1215–27. 21530003 10.1016/j.neurobiolaging.2011.02.012 PMC3179821

[38] van Arendonk J, Neitzel J, Steketee RME, van Assema DME, Vrooman HA, Segbers M, Diabetes and hypertension are related to amyloid-beta burden in the population-based Rotterdam Study. Brain. 2023;146(1):337–48. 36374264 10.1093/brain/awac354 PMC9825526

[39] Launer LJ, Ross GW, Petrovitch H, Masaki K, Foley D, White LR, Havlik RJ. Midlife blood pressure and dementia: the Honolulu-Asia aging study. Neurobiol Aging. 2000;21(1):49–55. 10794848 10.1016/s0197-4580(00)00096-8

[40] Israeli-Korn SD, Masarwa M, Schechtman E, Abuful A, Strugatsky R, Avni S, Hypertension increases the probability of Alzheimer’s disease and of mild cognitive impairment in an Arab community in northern Israel. Neuroepidemiology. 2010;34(2):99–105. 20016220 10.1159/000264828 PMC2855875

[41] Milà-Alomà M, Salvadó G, Gispert JD, Vilor-Tejedor N, Grau-Rivera O, Sala-Vila A, Amyloid beta, tau, synaptic, neurodegeneration, and glial biomarkers in the preclinical stage of the Alzheimer’s continuum. Alzheimers Dement. 2020;16(10):1358–71. 32573951 10.1002/alz.12131 PMC7586814

[42] Erickson P, Simrén J, Brum WS, Ennis GE, Kollmorgen G, Suridjan I, Prevalence and Clinical Implications of a β-Amyloid-Negative, Tau-Positive Cerebrospinal Fluid Biomarker Profile in Alzheimer Disease. JAMA Neurol. 2023;80(9):969–79. 37523162 10.1001/jamaneurol.2023.2338 PMC10391361

[43] Guo Y, Shen XN, Wang HF, Chen SD, Zhang YR, Chen SF, The dynamics of plasma biomarkers across the Alzheimer’s continuum. Alzheimers Res Ther. 2023;15(1):31. 36750875 10.1186/s13195-023-01174-0 PMC9906840

[44] Olsson A, Vanderstichele H, Andreasen N, De Meyer G, Wallin A, Holmberg B, Simultaneous measurement of beta-amyloid(1–42), total tau, and phosphorylated tau (Thr181) in cerebrospinal fluid by the xMAP technology. Clin Chem. 2005;51(2):336–45. 15563479 10.1373/clinchem.2004.039347

[45] Vanderstichele H, Bibl M, Engelborghs S, Le Bastard N, Lewczuk P, Molinuevo JL, Standardization of preanalytical aspects of cerebrospinal fluid biomarker testing for Alzheimer’s disease diagnosis: a consensus paper from the Alzheimer’s Biomarkers Standardization Initiative. Alzheimers Dement. 2012;8(1):65–73. 22047631 10.1016/j.jalz.2011.07.004

[46] Sharma RA, Varga AW, Bubu OM, Pirraglia E, Kam K, Parekh A, Obstructive Sleep Apnea Severity Affects Amyloid Burden in Cognitively Normal Elderly. A Longitudinal Study. Am J Respir Crit Care Med. 2018;197(7):933–43. 29125327 10.1164/rccm.201704-0704OC PMC6020410

[47] Kuznetsova A, Brockhoff P, Christensen RHB. lmerTest Package: Tests in Linear Mixed Effects Models.: Journal of Statistical Software; 2017.

[48] Yoshida K, Bartel A. tableone: Create ‘Table 1’ to Describe Baseline Characteristics with or without Propensity Score Weights. R package version 0.13.2.2022.

[49] Wickham H. ggplot2: Elegant Graphics for Data Analysis.: Springer-Verlag New York; 2016.

[50] Shaw LM, Vanderstichele H, Knapik-Czajka M, Clark CM, Aisen PS, Petersen RC, Cerebrospinal fluid biomarker signature in Alzheimer’s disease neuroimaging initiative subjects. Ann Neurol. 2009;65(4):403–13. 19296504 10.1002/ana.21610 PMC2696350

[51] Schröder S, Heck J, Groh A, Frieling H, Bleich S, Kahl KG, White Blood Cell and Platelet Counts Are Not Suitable as Biomarkers in the Differential Diagnostics of Dementia. Brain Sci. 2022;12(11). 10.3390/brainsci12111424 PMC9688654 36358351

[52] Li T, Wang C, Liu Y, Li B, Zhang W, Wang L, Neutrophil Extracellular Traps Induce Intestinal Damage and Thrombotic Tendency in Inflammatory Bowel Disease. J Crohns Colitis. 2020;14(2):240–53. 31325355 10.1093/ecco-jcc/jjz132

[53] Wright HL, Lyon M, Chapman EA, Moots RJ, Edwards SW. Rheumatoid Arthritis Synovial Fluid Neutrophils Drive Inflammation Through Production of Chemokines, Reactive Oxygen Species, and Neutrophil Extracellular Traps. Front Immunol. 2020;11:584116. 33469455 10.3389/fimmu.2020.584116 PMC7813679

[54] Liang X, Xiu C, Liu M, Lin C, Chen H, Bao R, Platelet-neutrophil interaction aggravates vascular inflammation and promotes the progression of atherosclerosis by activating the TLR4/NF-κB pathway. J Cell Biochem. 2019;120(4):5612–9. 30302814 10.1002/jcb.27844

[55] Krishnamoorthy N, Douda DN, Brüggemann TR, Ricklefs I, Duvall MG, Abdulnour RE, Neutrophil cytoplasts induce T(H)17 differentiation and skew inflammation toward neutrophilia in severe asthma. Sci Immunol. 2018;3(26). 10.1126/sciimmunol.aao4747 PMC6320225 30076281

[56] Baik SH, Cha MY, Hyun YM, Cho H, Hamza B, Kim DK, Migration of neutrophils targeting amyloid plaques in Alzheimer’s disease mouse model. Neurobiol Aging. 2014;35(6):1286–92. 24485508 10.1016/j.neurobiolaging.2014.01.003 PMC4248665

[57] Park J, Baik SH, Mook-Jung I, Irimia D, Cho H. Mimicry of Central-Peripheral Immunity in Alzheimer’s Disease and Discovery of Neurodegenerative Roles in Neutrophil. Front Immunol. 2019;10:2231. 31611872 10.3389/fimmu.2019.02231 PMC6776120

[58] Scali C, Prosperi C, Bracco L, Piccini C, Baronti R, Ginestroni A, Neutrophils CD11b and fibroblasts PGE(2) are elevated in Alzheimer’s disease. Neurobiol Aging. 2002;23(4):523–30. 12009501 10.1016/s0197-4580(01)00346-3

[59] Dong Y, Lagarde J, Xicota L, Corne H, Chantran Y, Chaigneau T, Neutrophil hyperactivation correlates with Alzheimer’s disease progression. Ann Neurol. 2018;83(2):387–405. 29369398 10.1002/ana.25159

[60] Martínez de Toda I, Vida C, Díaz-Del Cerro E, De la Fuente M. The Immunity Clock. J Gerontol A Biol Sci Med Sci. 2021;76(11):1939–45. 33979432 10.1093/gerona/glab136

[61] Nogueira-Neto J, Cardoso AS, Monteiro HP, Fonseca FL, Ramos LR, Junqueira VB, Simon KA. Basal neutrophil function in human aging: Implications in endothelial cell adhesion. Cell Biol Int. 2016;40(7):796–802. 27109745 10.1002/cbin.10618

[62] Wenisch C, Patruta S, Daxböck F, Krause R, Hörl W. Effect of age on human neutrophil function. J Leukoc Biol. 2000;67(1):40–5. 10647996 10.1002/jlb.67.1.40

[63] Blasko I, Marx F, Steiner E, Hartmann T, Grubeck-Loebenstein B. TNFalpha plus IFNgamma induce the production of Alzheimer beta-amyloid peptides and decrease the secretion of APPs. Faseb j. 1999;13(1):63–8. 9872930 10.1096/fasebj.13.1.63

[64] Michelucci A, Heurtaux T, Grandbarbe L, Morga E, Heuschling P. Characterization of the microglial phenotype under specific pro-inflammatory and anti-inflammatory conditions: Effects of oligomeric and fibrillar amyloid-beta. J Neuroimmunol. 2009;210(1–2):3–12. 19269040 10.1016/j.jneuroim.2009.02.003

[65] Yamamoto M, Kiyota T, Walsh SM, Liu J, Kipnis J, Ikezu T. Cytokine-mediated inhibition of fibrillar amyloid-beta peptide degradation by human mononuclear phagocytes. J Immunol. 2008;181(6):3877–86. 18768842 10.4049/jimmunol.181.6.3877 PMC2603577

[66] Kitazawa M, Cheng D, Tsukamoto MR, Koike MA, Wes PD, Vasilevko V, Blocking IL-1 signaling rescues cognition, attenuates tau pathology, and restores neuronal β-catenin pathway function in an Alzheimer’s disease model. J Immunol. 2011;187(12):6539–49. 22095718 10.4049/jimmunol.1100620 PMC4072218

[67] Wang Y, Jin S, Sonobe Y, Cheng Y, Horiuchi H, Parajuli B, Interleukin-1β induces blood-brain barrier disruption by downregulating Sonic hedgehog in astrocytes. PLoS One. 2014;9(10):e110024. 25313834 10.1371/journal.pone.0110024 PMC4196962

[68] Sheng JG, Zhu SG, Jones RA, Griffin WS, Mrak RE. Interleukin-1 promotes expression and phosphorylation of neurofilament and tau proteins in vivo. Exp Neurol. 2000;163(2):388–91. 10833312 10.1006/exnr.2000.7393 PMC3886634

[69] Ising C, Venegas C, Zhang S, Scheiblich H, Schmidt SV, Vieira-Saecker A, NLRP3 inflammasome activation drives tau pathology. Nature. 2019;575(7784):669–73. 31748742 10.1038/s41586-019-1769-z PMC7324015

[70] Yang H, Park SY, Baek H, Lee C, Chung G, Liu X, Adoptive therapy with amyloid-β specific regulatory T cells alleviates Alzheimer’s disease. Theranostics. 2022;12(18):7668–80. 36451854 10.7150/thno.75965 PMC9706584

[71] Machhi J, Yeapuri P, Lu Y, Foster E, Chikhale R, Herskovitz J, CD4+ effector T cells accelerate Alzheimer’s disease in mice. J Neuroinflammation. 2021;18(1):272. 34798897 10.1186/s12974-021-02308-7 PMC8603581

[72] Chen X, Firulyova M, Manis M, Herz J, Smirnov I, Aladyeva E, Microglia-mediated T cell infiltration drives neurodegeneration in tauopathy. Nature. 2023;615(7953):668–77. 36890231 10.1038/s41586-023-05788-0 PMC10258627

[73] Zhang J, Ke KF, Liu Z, Qiu YH, Peng YP. Th17 cell-mediated neuroinflammation is involved in neurodegeneration of aβ1–42-induced Alzheimer’s disease model rats. PLoS One. 2013;8(10):e75786. 24124514 10.1371/journal.pone.0075786 PMC3790825

[74] Yang YM, Shang DS, Zhao WD, Fang WG, Chen YH. Microglial TNF-α-dependent elevation of MHC class I expression on brain endothelium induced by amyloid-beta promotes T cell transendothelial migration. Neurochem Res. 2013;38(11):2295–304. 23990225 10.1007/s11064-013-1138-5

[75] Fani L, Georgakis MK, Ikram MA, Ikram MK, Malik R, Dichgans M. Circulating biomarkers of immunity and inflammation, risk of Alzheimer’s disease, and hippocampal volume: a Mendelian randomization study. Transl Psychiatry. 2021;11(1):291. 34001857 10.1038/s41398-021-01400-z PMC8129147

[76] Xu H, Jia J. Single-Cell RNA Sequencing of Peripheral Blood Reveals Immune Cell Signatures in Alzheimer’s Disease. Front Immunol. 2021;12:645666. 34447367 10.3389/fimmu.2021.645666 PMC8382575

[77] Man SM, Ma YR, Shang DS, Zhao WD, Li B, Guo DW, Peripheral T cells overexpress MIP-1alpha to enhance its transendothelial migration in Alzheimer’s disease. Neurobiol Aging. 2007;28(4):485–96. 16600437 10.1016/j.neurobiolaging.2006.02.013

[78] Bellaver B, Povala G, Ferreira PCL, Ferrari-Souza JP, Leffa DT, Lussier FZ, Astrocyte reactivity influences amyloid-β effects on tau pathology in preclinical Alzheimer’s disease. Nat Med. 2023;29(7):1775–81. 37248300 10.1038/s41591-023-02380-x PMC10353939

[79] Rabin JS, Nichols E, La Joie R, Casaletto KB, Palta P, Dams-O’Connor K, Cerebral amyloid angiopathy interacts with neuritic amyloid plaques to promote tau and cognitive decline. Brain. 2022;145(8):2823–33. 35759327 10.1093/brain/awac178 PMC9420012

[80] Milà-Alomà M, Ashton NJ, Shekari M, Salvadó G, Ortiz-Romero P, Montoliu-Gaya L, Plasma p-tau231 and p-tau217 as state markers of amyloid-β pathology in preclinical Alzheimer’s disease. Nat Med. 2022;28(9):1797–801. 35953717 10.1038/s41591-022-01925-w PMC9499867

[81] Suárez-Calvet M, Karikari TK, Ashton NJ, Lantero Rodríguez J, Milà-Alomà M, Gispert JD, Novel tau biomarkers phosphorylated at T181, T217 or T231 rise in the initial stages of the preclinical Alzheimer’s continuum when only subtle changes in Aβ pathology are detected. EMBO Mol Med. 2020;12(12):e12921. 33169916 10.15252/emmm.202012921 PMC7721364

[82] Dong G, Gan M, Xu S, Xie Y, Zhou M, Wu L. The neutrophil-lymphocyte ratio as a risk factor for all-cause and cardiovascular mortality among individuals with diabetes: evidence from the NHANES 2003–2016. Cardiovasc Diabetol. 2023;22(1):267. 37775767 10.1186/s12933-023-01998-y PMC10541705

[83] ong X, Lu Z, Feng X, Yu C, Xue M, Yu L, Elevated Neutrophil-to-Lymphocyte Ratio Predicts Depression After Intracerebral Hemorrhage. Neuropsychiatr Dis Treat. 2020;16:2153–9. 33061386 10.2147/NDT.S269210 PMC7518785

[84] Iaciu CI, Emilescu RA, Cotan HT, Nitipir C. Systemic Neutrophil-to-Lymphocyte Ratio as a Prognostic Biomarker for Colon Cancer. Chirurgia (Bucur). 2023;118(3):260–71. 37480352 10.21614/chirurgia.2023.v.118.i.3.p.260

[85] Şahin A, Toprak T, Kutluhan MA, Vural Y, Ürkmez A, Verit A. Increased neutrophil/lymphocyte ratio in testicular cancer. Arch Ital Urol Androl. 2019;91(2). 10.4081/aiua.2019.2.97 31266274

[86] Tutan D, Erdoğan Kaya A, Eser B. The relationship between neutrophil lymphocyte ratio, platelet lymphocyte ratio, and depression in dialysis patients. Medicine (Baltimore). 2023;102(37):e35197. 37713848 10.1097/MD.0000000000035197 PMC10508398

[87] Reimand J, Collij L, Scheltens P, Bouwman F, Ossenkoppele R. Association of amyloid-β CSF/PET discordance and tau load 5 years later. Neurology. 2020;95(19):e2648–e57. 32913020 10.1212/WNL.0000000000010739 PMC7963352

